# Motor Exit Point (MEP) Glia: Novel Myelinating Glia That Bridge CNS and PNS Myelin

**DOI:** 10.3389/fncel.2018.00333

**Published:** 2018-10-02

**Authors:** Laura Fontenas, Sarah Kucenas

**Affiliations:** Department of Biology, University of Virginia, Charlottesville, VA, United States

**Keywords:** myelin, oligodendrocyte, schwann cell, motor exit point glia, zebrafish, boundary cap cell

## Abstract

Oligodendrocytes (OLs) and Schwann cells (SCs) have traditionally been thought of as the exclusive myelinating glial cells of the central and peripheral nervous systems (CNS and PNS), respectively, for a little over a century. However, recent studies demonstrate the existence of a novel, centrally-derived peripheral glial population called motor exit point (MEP) glia, which myelinate spinal motor root axons in the periphery. Until recently, the boundaries that exist between the CNS and PNS, and the cells permitted to cross them, were mostly described based on fixed histological collections and static lineage tracing. Recent work in zebrafish using *in vivo*, time-lapse imaging has shed light on glial cell interactions at the MEP transition zone and reveals a more complex picture of myelination both centrally and peripherally.

## Introduction

Myelin is a unique plasma membrane extension produced by specialized glial cells that is wrapped around axons and facilitates rapid transmission of electrical impulses over long distances. Although myelin appeared 430 million years ago in the vertebrate lineage (Gould et al., 2008; Zalc, 2016), continuous efforts from various fields of biology are still unraveling the many mysteries of myelin and myelin producing cells.

The nervous system is a complex organ whose function depends on the precise organization of its components. The central nervous system (CNS) consists of the brain and the spinal cord, while the peripheral nervous system (PNS) consists of neural tissue outside of the CNS, including motor and sensory axons and sensory neurons clustered in ganglia. While many neurons establish circuits within the CNS, some of these cells send their axons (e.g., motor axons) freely across specialized CNS boundaries known as transition zones (TZs) and travel long distances throughout the organism and ultimately make synapses on peripheral tissues, including muscle. By associating tightly with axons to ultimately ensheath and insulate them with fatty membranes called myelin, myelinating cells increase nerve impulse conduction speed and strengthen the function and efficiency of the whole nervous system.

In the CNS, oligodendrocytes (OLs), which are derived from neural tube precursors, make myelin in the brain and spinal cord, while Schwann cells (SCs), which originate from the neural crest (NC), myelinate axons of the PNS (Jessen and Mirsky, 2005; Bergles and Richardson, 2015). In addition to expressing different sets of genes, central and peripheral glia also ensheath axons in myelin in distinct ways. OLs expand multiple membrane processes, which wrap several axonal segments that often belong to different neurons, while a single SC ensheaths a single axonal segment. Although OLs and SCs myelinate axons in mechanistically distinct ways, their territories come into close proximity along common axons at motor exit point (MEP) TZs, where they are separated by only a few microns (Fraher and Kaar, 1984; Fraher, 2002).

**Figure 1:**
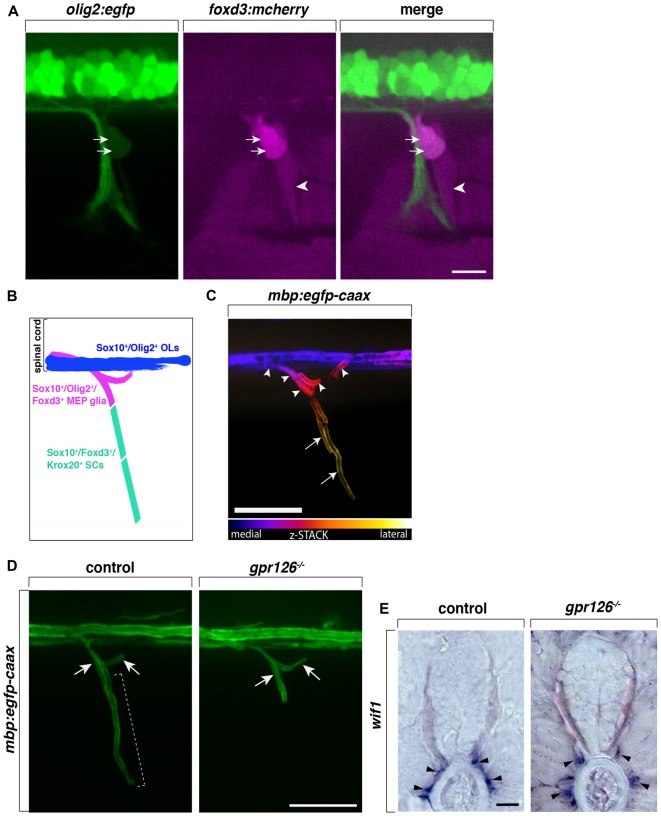
Centrally-derived motor exit point (MEP) glia myelinate motor nerve roots. **(A)** Lateral view of a *olig2:egfp;foxd3:mcherry* zebrafish trunk showing *olig2*^+^/*foxd3*^+^ MEP glia (arrows) and *olig2*^−^/*foxd3*^+^ SCs (arrowheads) along a motor nerve at 55 hour post-fertilization (hpf). **(B)** Diagram representing central and peripheral myelinating glial markers. **(C)** Pseudo-colored z-stack of a lateral view of a *mbp*:*egfp-caax* trunk at 5 day post-fertilization (dpf). MEP glial myelin sheaths (arrowheads) originate within the spinal cord and project laterally along motor nerve axons, coming close to schwann cells (SC) myelin (arrows). **(D)** SC myelin (bracket) but not MEP glial myelin (arrows) is absent in G-protein coupled receptor 126 (*gpr126*) mutants at 5 days post fertilization (dpf). **(E)** *In situ* hybridization showing the presence of *Wnt-inhibitory factor 1* (*wif1*^+^) MEP glia (arrowheads) along motor nerve roots in 3 dpf control and *gpr126* mutant larvae. Scale bar, **(A–D)** 50 μm, **(E)** 20 μm.

The mammalian CNS/PNS boundary is delineated by a thick layer of astrocytic endfeet that is continuous with the glia limitans which covers the surface of the spinal cord with the exception of perforations that allow axons to cross (reviewed in Fontenas and Kucenas, 2017). Ultrastructural studies show that central and peripheral glial cells, namely OLs and SCs, are present near the MEP TZ, but are never seen intermixing. In fact, MEP TZs are described as an abrupt transition between central and peripheral glia (Doucette, 1991; Franklin and Blakemore, 1993; Fraher et al., 2007). However, intriguingly, using *in vivo* imaging in zebrafish, we and others observe the presence of continuous *mbp*^+^ myelin internodes along motor axons crossing the MEP TZ (Figure 1C; Monk et al., 2009; Almeida et al., 2011; Auer et al., 2018).

The two distinct yet connected halves of the nervous system have their own, non-overlapping myelin forming cells and myelin sheaths. Does this mean myelinating glia cannot function in the half where they do not originate? What keeps them segregated in the developing and mature nervous system? How does unique myelin on either side of the CNS/PNS TZ result in an efficiently insulated nerve?

Work from our lab and others demonstrates the presence of additional glial cells outside the spinal cord at the TZ between the CNS and the PNS, which function to segregate central and peripheral components from ectopically crossing this boundary (Vermeren et al., 2003; Kucenas et al., 2009; Coulpier et al., 2010; Smith et al., 2014). While most studies previously focused on nervous system TZs using histological and ultrastructural methods, recent studies using *in vivo*, time-lapse imaging in zebrafish have shed light on these dynamic structures and revealed novel glial cell populations that cross these TZs (Kucenas et al., 2008, 2009; Smith et al., 2014; Welsh and Kucenas, 2018).

In this article, we will provide a short review on MEP glia, a recently discovered population of centrally-derived glial cells that myelinate spinal motor nerve root axons and compare them to traditional myelinating glial populations in both form and function.

## MEP Glia Are Centrally-Derived Peripheral Myelinating Cells

Until recently, the TZs between the CNS and the PNS were thought to be selectively permeable to axons either entering or exiting the spinal cord, while the establishment and maintenance of the territories occupied by glial cells remained poorly described and understood. However, recent studies demonstrate that MEP TZs are occupied by highly dynamic cell populations and are precisely regulated over the course of nervous system development. While OL lineage cells and SCs segregate and function in the CNS and PNS, respectively, other glial cell populations freely cross the MEP TZ as spinal motor nerves are being formed (Kucenas et al., 2008, 2009; Smith et al., 2014, 2016; Fontenas and Kucenas, 2017).

One of these cell populations, MEP glia, originate from pMN domain precursors in the ventral neural tube, which also give rise to motor neurons (MNs) and OL lineage cells. After exiting the CNS, MEP glia reside just outside of the ventral spinal cord along spinal motor nerve root axons, occupying axonal territory between SCs in the periphery and OLs in the spinal cord. The pMN domain constitutes the major site of expression of the basic helix-loop-helix (bHLH) transcription factor Olig2. In zebrafish, *olig2^+^* precursors first produce MNs and then switch to generate glial cells at approximately 36 hours post fertilization (hpf; Smith et al., 2014; Ravanelli and Appel, 2015). MEP glia exit the ventral spinal cord through the MEP TZ at around 50 hpf, before the onset of oligodendrocyte progenitor cell (OPC) migration, and then divide to populate the motor root.

MEP glia and OL lineage cells share a common progenitor and as a consequence, the two myelinating cell populations—although they function in two distinct halves of the nervous system—share common markers. MEP glia express *olig2* (Figures 1A,B), whose expression is progressively turned off after MEP glia reach the motor nerve root, prior to motor axon myelination (Smith et al., 2014). Both central and peripheral myelinating glial cells in zebrafish as well as in mammals, express the transcription factor Sox10 (Figure 1B). Numerous studies show that sox10 is required for NC cell development, including SCs, as well as for OL lineage differentiation and promotes myelin gene expression (Kelsh and Eisen, 2000; Dutton et al., 2001; Gilmour et al., 2002; Kucenas et al., 2009; Takada et al., 2010). Unsurprisingly, MEP glia also express and require sox10 for their development and function (Kucenas et al., 2009; Smith et al., 2014). *In vivo*, time-lapse imaging has recently revealed sox10 expression in MEP glia before they even exit the spinal cord and is maintained throughout their development and differentiation. The pioneer study demonstrating the existence of centrally-derived glial cells at the MEP TZ, shows that zebrafish *colorless* (*cls*) mutants, which harbor a mutation in sox10 and lack SCs, have peripheral OPCs that differentiate and myelinate spinal motor nerves, suggesting that sox10 may be required for MEP glial function or survival (Kucenas et al., 2009).

Intriguingly, MEP glia not only express the transcription factors *sox10* and *olig2*, which together are a combination that is characteristic of CNS myelinating glia, they also express *foxd3* (Figures 1A,B), a transcription factor present in all NC cells including sox10^+^ peripheral glia such as SCs in zebrafish (Odenthal and Nüsslein-Volhard, 1998; Gilmour et al., 2002; Hochgreb-Hägele and Bronner, 2013; Smith et al., 2014). Using *in vivo*, time-lapse imaging and the *Gt(foxd3:mcherry)* transgenic line that faithfully mimics the endogenous expression of *foxd3* (Hochgreb-Hägele and Bronner, 2013),  *foxd3* expression was observed as early as 46 hpf, before MEP glia exit the spinal cord, and persisted in these cells as late as 8 day post-fertilization (dpf; Smith et al., 2014). Despite the fact that they express the peripheral marker *foxd3*, MEP glia do not express krox20 (also known as early growth response protein 2—egr2b), a transcription factor expressed by boundary cap (BC) cells that is also required for SC myelination (data not shown; Wilkinson et al., 1989; Topilko et al., 1994; Monk et al., 2009; Coulpier et al., 2010; Smith et al., 2014).

In chick and mouse, BC cells are traditionally described as NC derivatives that migrate along the ventral path and transiently reside in clusters at dorsal root entry zone (DREZ) and MEP TZs, where axons enter and exit the spinal cord, respectively (Niederländer and Lumsden, 1996; Golding and Cohen, 1997; Vermeren et al., 2003). BC cells eventually give rise to neuronal and glial cell populations, including myelinating SCs (Maro et al., 2004; Gresset et al., 2015; Radomska and Topilko, 2017). At the MEP, BC cells function to constrain MN cell bodies to the spinal cord, as their ablation leads to ectopic MNs along motor nerve roots (Vermeren et al., 2003; Bron et al., 2007). The Topilko laboratory also demonstrated that upon Krox20 inactivation, which results in the absence of both SCs and BC cells, OL ectopically populate the ventral and dorsal roots (Coulpier et al., 2010).

To date, BC cells have not been described in zebrafish, however, MEP glia do express the BC cell marker *Wnt-inhibitory factor 1 (wif1*; Figure 1E), which no other myelinating cells express (Coulpier et al., 2009; Smith et al., 2014). Taken together, MEP glia express a subset of both central and peripheral glial markers (*sox10*, *olig2*, *foxd3* and *wif1*) that are not found all together in any other myelinating glial cell type (Figure 1B). Although MEP glia originate in the CNS and function in the PNS, their identity is not truly that of either a central or a peripheral glial cell, but rather appear to be a hybrid glial cell.

Previous work from our lab shows that MEP glia differentiate into myelinating glia and by 3 dpf, start to ensheath the proximal portion of spinal motor nerve axons, also known as motor nerve roots (Figures 1C,D; Smith et al., 2014). In the same initial study, we demonstrate that MEP glia restrict OPCs to the spinal cord through contact-mediated repulsion. Using *in vivo*, time-lapse imaging, we describe that prior to 3 dpf, OPCs extend membrane processes into the periphery to sense the environment. Immediately upon contact with a MEP glial cell, OPCs retract their processes from the periphery and pursue their migration within the spinal cord. Under normal physiological conditions, OPC cell bodies are never found outside of the spinal cord but invade peripheral nerves in the absence of MEP glia (Kucenas et al., 2009; Smith et al., 2014). Consistent with these findings, OPCs exit the spinal cord through MEP TZs in mutants lacking all peripheral myelinating glia (Smith et al., 2014). For example, OPCs are found along peripheral nerves in *erbb3b* mutants that harbor a mutation in the receptor tyrosine kinase, erbb3b and lack all peripheral myelinating glia (Lyons et al., 2005; Smith et al., 2014; Morris et al., 2017). Additionally, using *in vivo*, time-lapse imaging coupled with single cell ablation using a nitrogen-pulsed laser, our lab showed that specific ablation of MEP glia leads to the ectopic exit of OPCs from the spinal cord (Smith et al., 2014). However, OPCs are never found in the PNS when regions surrounding MEP glia are ablated or when radial glia, another glial cell population present at the MEP TZ, are genetically ablated (Smith et al., 2014, 2016). These observations demonstrate that MEP glia contribute to a selective gating mechanism at MEP TZs. Direct interactions between myelinating glial cells from the CNS and PNS participate in regulating glial migration across TZs and contribute to the establishment and maintenance of the CNS/PNS boundary and continuous myelination along spinal motor axons.

## Elucidating the Molecular Mechanisms That Mediate MEP Glial Development and Function

Although myelin promotes the rapid propagation of electrical activity, not all axons are myelinated. In the PNS, studies unanimously point to a straightforward mechanism, whereby axonal caliber determines whether or not myelination will occur and also determines the extent of myelin produced (Taveggia et al., 2005; Newbern and Birchmeier, 2010; Perlin et al., 2011). On the other hand, central myelination is a divisive topic, and although we still understand only a little about how neuronal activity influences OL myelination, coupling neuronal activity to myelination arouses interest and has become a rapidly growing field. Elegant work from the Chan laboratory demonstrates that OLs can myelinate electrically silent nanofibers *in vitro* (Lee et al., 2012). However, there are other studies that demonstrate that environmental experience and neuronal activity *in vivo* are essential for myelination. Evidence from studies using *in vitro* models, *in vivo*, time-lapse imaging in zebrafish, and also mouse models suggest that OL lineage cells can detect and respond to extrinsic cues during their development and differentiation, but the source and identity of these signals are matter of debate (Kirby et al., 2006; Hines et al., 2015; Mensch et al., 2015; Etxeberria et al., 2016; Koudelka et al., 2016; see; Almeida and Lyons, 2017; Welsh and Kucenas, 2018 for recent reviews).

**Figure 2:**
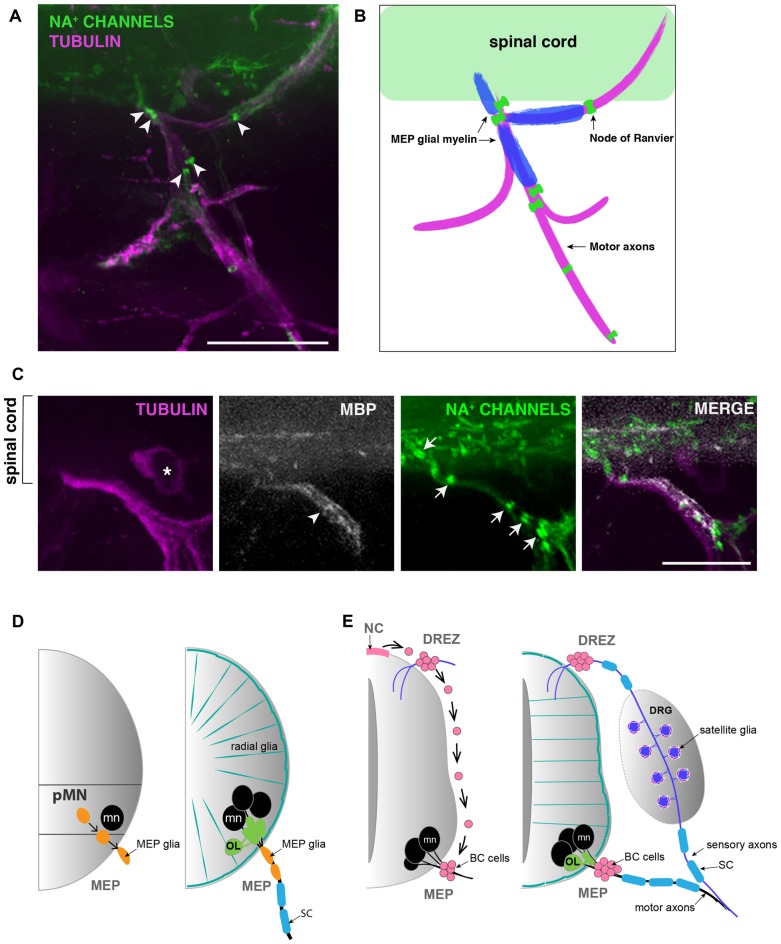
MEP glial myelin is flanked by nodes of ranvier. **(A)** Lateral view of a 5 dpf zebrafish trunk stained with antibodies specific to pan Na^+^ channels (clone k58/35) and acetylated tubulin shows that MEP glial territory is flanked by sodium channels clustered in Nodes of Ranvier (arrowheads) along motor nerve roots. **(B)** Diagram showing MBP^+^ MEP glial myelin (blue) delineated by nodes of Ranvier (green) along motor nerve root axons (magenta). **(C)** Immunostaining showing MBP^+^ MEP glial myelin (arrowhead) and nodes of Ranvier (arrows) along motor nerve root axons (magenta). Asterisks point to dorsal root ganglion (DRG). **(D)** Schematic of zebrafish MEP transition zones (TZs) and the diverse populations of glial cells orchestrating the CNS/PNS boundary. CNS-derived MEP glia (orange) that reside along motor neuron (mn; black) axons restrict the oligodendrocyte lineage (OL; green) to the spinal cord. Radial glia (teal green) cover the surface of the spinal cord and prevent peripheral glia such as MEP glia and SC (blue) from entering the CNS. **(E)** Schematic of a mammalian neural tube showing neural crest (NC)-derived boundary cap (BC) cells (pink) sitting at the dorsal root entry zone (DREZ) and MEP TZs. BC cells prevent mn (black) cell bodies from transgressing the spinal cord boundary. Scale bar, **(A)** 50 μm, **(C)** 25 μm.

MEP glia ensheath and myelinate motor axon segments that do not overlap with myelin internodes made by SCs found further distally along the nerve. In fact, the boundary between MEP glial and SC myelin sheaths is clearly delineated by a node of Ranvier (Figures 2A,B). Like the well described and century-old myelinating glial cells SCs and OLs, MEP glia have the ability to cluster sodium channels to nodes of Ranvier as they initiate axonal wrapping, thus promoting saltatory conduction from the very proximal portion of motor nerve root axons (Figures 2A–C; Voas et al., 2009). MEP glial myelin internodes are flanked by nodes of Ranvier inside the spinal cord and just peripheral to the MEP TZ (Figures 2A–C). MEP glia also express *myelin basic protein (mbp)* and both *mbp* and transcript and MBP protein can be detected along motor nerve root axons as early as 4 dpf (Figures 2B,C; Brösamle and Halpern, 2002; Smith et al., 2014).

Fascinatingly, in *G protein-coupled receptor 126* (*gpr126*) mutants, where SCs are arrested at the pro-myelinating stage and fail to myelinate peripheral axons, Monk et al. (2009) described the presence of myelin along spinal motor root axons, reminiscent of projections of central myelin through MEP TZs. At the time, MEP glia were yet to be discovered and characterized. Therefore, projections of central myelin were the most plausible explanation for such a phenotype. However, recent work from our lab has extended these findings and we now know that the projections of “central myelin” into the periphery observed in *gpr126* mutants are, in fact, MEP glial myelin sheaths (Figures 1D,E). These data therefore demonstrate that, unlike SCs, MEP glia do not require *gpr126* to initiate myelination. And as previously mentioned, these glial cells also do not express Krox20. Therefore, they are a CNS-derived, peripheral glial cell that myelinates peripheral axons, but uses a distinct molecular mechanism to initiate myelination.

## Are MEP Glia the Only Centrally-Derived Peripheral Glia?

For years, mammalian BC cells were thought to constrain MN cell bodies to the spinal cord by repulsion from outside the spinal cord (Bron et al., 2007; Mauti et al., 2007; Chauvet and Rougon, 2008; Bravo-Ambrosio and Kaprielian, 2011; Garrett et al., 2016). However, how this physically worked was unclear, as neither BC cells or MN cell bodies were close enough to physically interact to mediate the repulsion (Figure 2E). Intriguingly, a recent review from the Topilko laboratory revealed a new piece of data that shows that *Krox20^+^* BC cells project membrane processes across the MEP TZ into the ventral spinal cord (Radomska and Topilko, 2017). These recent data are consistent with our longstanding hypothesis that BC cells might be a diverse cell population and include a subpopulation of centrally-derived cells, analogous to zebrafish MEP glia (Figure 2D). Consistent with this hypothesis, a recent study demonstrates that in a demyelination context, Olig2^+^ cells residing within the mouse spinal cord give rise to SCs, which are usually NC-derived (Zawadzka et al., 2010). Is this phenomenon a response to demyelination, or does it also exist under normal circumstances? Previous studies describe the identification of perineurial glia, the glial cells that form the perineurium found around peripheral motor nerves, and demonstrate that they are CNS-derived in *Drosophila*, zebrafish and mouse (Sepp et al., 2001; Kucenas et al., 2008; Clark et al., 2014; Kucenas, 2015). However, the origin of perineurial glia was of intense debate for decades as they were previously thought to be fibroblasts. Could this model of CNS-derived peripheral glia apply to other cell types? Understanding the diversity of origin for glial cells and their ability to be specified in one region of the nervous system but function in another, could provide insight into cell interaction, segregation and selective gating mechanisms.

## Conclusion

MEP glial development and function raise many new questions. What are the signals mediating OPC-MEP glial interactions across the MEP TZ? Do MEP glia also interact with SCs and via which molecular mechanisms? Does this novel, centrally-derived glial cell population adopt a central-like myelination pattern, involving the extension of multiple membrane processes to wrap more than one axonal segment? Or does it behave more like a Schwann cell, ensheathing a single axonal segment in a truly peripheral-like fashion? Or alternatively, is MEP glial myelin a kind of hybrid myelin featuring both central and peripheral characteristics? How do MEP glia myelinate if they do not require Krox20 or Gpr126? Electron microscopy of the MEP TZ will be needed to examine MEP glial development at the ultrastructural level to characterize their myelin sheaths that connect the CNS and PNS. RNA-sequencing coupled to genome editing tools such as CRISPR/Cas9 will shed light on the molecular mechanisms that drive MEP glial development, their gating function and differentiation into peripheral myelinating glia. Recent studies in zebrafish have uncovered the existence of novel centrally-derived myelinating glia that function in the PNS and are required for nervous system TZ integrity, and future work may change our way of thinking about mammalian myelinating glia as well.

## Author Contributions

LF wrote the manuscript with input from SK.

## Conflict of Interest Statement

The authors declare that the research was conducted in the absence of any commercial or financial relationships that could be construed as a potential conflict of interest.

## Abbreviations

BC: boundary cap
CNS: central nervous system
dpf: day post-fertilization
DREZ: dorsal root entry zone
DRG: dorsal root ganglion
eGFP: enhanced green fluorescent protein
Gpr126: G-protein coupled receptor 126
hpf: hour post-fertilization
MBP: myelin basic protein
MEP: motor exit point
MN: motor neuron
NC: neural crest
OL: oligodendrocyte
OPC: oligodendrocyte progenitor cell
PNS: peripheral nervous system
SC: Schwann cell
TZ: transition zone
Wif1: Wnt-inhibitory factor 1.
